# Simultaneously Hermaphroditic Shrimp Use Lipophilic Cuticular Hydrocarbons as Contact Sex Pheromones

**DOI:** 10.1371/journal.pone.0017720

**Published:** 2011-04-20

**Authors:** Dong Zhang, John A. Terschak, Maggy A. Harley, Junda Lin, Jörg D. Hardege

**Affiliations:** 1 East China Sea Fisheries Research Institute, China Academy of Fisheries, Shanghai, People's Republic of China; 2 Department of Biological Sciences, The University of Hull, Hull, United Kingdom; 3 Vero Beach Marine Laboratory, Florida Institute of Technology, Vero Beach, Florida, United States of America; University of Plymouth, United Kingdom

## Abstract

Successful mating is essentially a consequence of making the right choices at the correct time. Animals use specific strategies to gain information about a potential mate, which is then applied to decision-making processes. Amongst the many informative signals, odor cues such as sex pheromones play important ecological roles in coordinating mating behavior, enabling mate and kin recognition, qualifying mate choice, and preventing gene exchange among individuals from different populations and species. Despite overwhelming behavioral evidence, the chemical identity of most cues used in aquatic organisms remains unknown and their impact and omnipresence have not been fully recognized. In many crustaceans, including lobsters and shrimps, reproduction happens through a cascade of events ranging from initial attraction to formation of a mating pair eventually leading to mating. We examined the hypothesis that contact pheromones on the female body surface of the hermaphroditic shrimp *Lysmata boggessi* are of lipophilic nature, and resemble insect cuticular hydrocarbon contact cues. Via chemical analyses and behavioural assays, we show that newly molted euhermaphrodite-phase shrimp contain a bouquet of odor compounds. Of these, (Z)-9-octadecenamide is the key odor with hexadecanamide and methyl linoleate enhancing the bioactivity of the pheromone blend. Our results show that in aquatic systems lipophilic, cuticular hydrocarbon contact sex pheromones exist; this raises questions on how hydrocarbon contact signals evolved and how widespread these are in the marine environment.

## Introduction

In species where mating competition is significant and the window of opportunity to mate is limited, sexual partners need to develop signals that can be used to make an informed decision on whether to invest in mating efforts [1], [2]. A male shrimp, for example, can only mate for a short period after the female moult; this precise moment is detected via a primary sensory signal, a sex pheromone. To reduce incidents of mistakes and their associated costs, many species utilize complex odor bouquets and often combine them with an established cascade of behaviours that encode a stereotyped response pattern [2], [3]. Behaviour cascades can include distinctive steps such as attracting a mate, a pre-mating ritual, and eventual mating, itself [4]. Contact cues, signals that are bound to the surface of the sender's body, represent the final decisive hurdle in the initiation of mating behaviour [5] and have been described to exist in many insect species [6], [7] as well as in crustaceans [8].

Two principle classifications of sex pheromones exist: distance cues and contact cues, and examples have been identified in an ever-growing number of species. In terrestrial animals, distance pheromones are usually air transmitted and, as such, are volatile compounds, while contact pheromones are coated on the body surface [8] requiring physical tactile interaction. In aquatic animals, such as crustaceans, distance pheromones are hypothesized as mostly polar, water-soluble compounds to enable transmission in odor plumes [4]. Contact pheromones should be relatively insoluble in water (non-polar) to remain on the exterior surfaces reducing the need to be replaced constantly. In decapod crustaceans, such as crabs [9], [10], lobsters [11], and crayfish [12], the urine of females contains a pheromone that acts over a distance to attract male mating partners. In caridean shrimp, contact pheromones have been proposed in mate recognition [13], [14] but the chemical identity of the compound(s) responsible remains unknown [8].

In the identification of crustacean contact sex pheromones, it is beneficial to note the analogous finding that such cues are well known in insects and have been chemically characterized as cuticular hydrocarbons (CHC's) [6]. In a variety of insect species, including Coleoptera, Lepidoptera, Hymenoptera and Diptera [5], [7], [8], [15], these CHC's function as inter- and intra-specific chemical signals. For example, contact pheromones exist for the fruit fly (both *Drosophila melanogaster* and *D. serrata*) [5] and serve to highlight the complexity of the chemical dialogue that takes place between the sexes. As recently described by Billeter et al., CHC's can carry unexpectedly diverse information including the sender's sex, age, physiological state, fitness, genetic trait, or mating status [16]. As such, CHC's play important roles in the evolution of signal traits and mating preferences [17].

The best studied contact pheromone systems in marine crustaceans are the harpacticoid copepods where glycoproteins were proposed as contact cues in *Tigriopus japonicus* [18]. In barnacles, *Balanus amphitrite*, gregarious larval settlement cues that are partially surface bound are protein complexes that enable species recognition at settlement [19]. Surface glycoproteins are potentially involved in mate recognition in caridean shrimp, *Palaemonetes pugio*, as recently demonstrated through competitive inhibition studies [20]; however, glycoproteins are not the contact pheromones in *Lysmata* shrimp [21].

**Figure 1:**
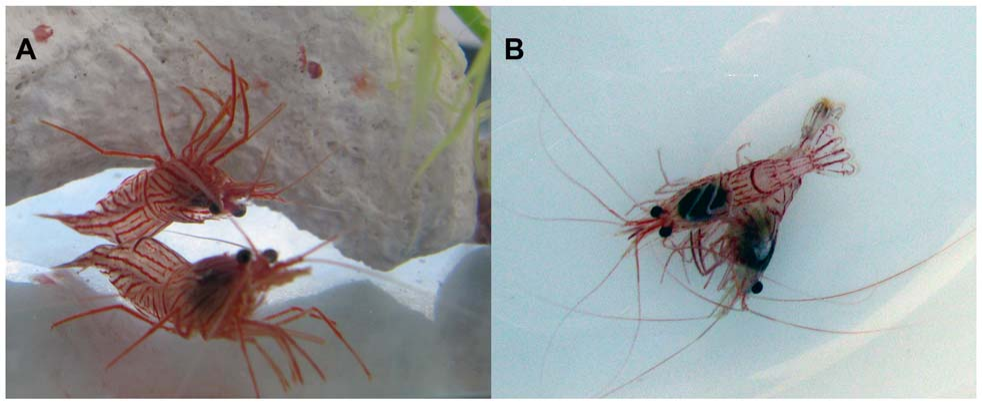
Sympatric simultaneous hermaphroditic shrimp, *Lysmata boggessi*. A: mature individuals, B: Reproductive behaviour with male grasping female.

In this study, we examined the hypothesis that contact pheromones on the female body surface of the hermaphroditic shrimp *Lysmata boggessi* are lipophilic in nature, and as such, resemble insect cuticular hydrocarbon contact cues [1], [6]. We use recent behavioral evidence that suggests the existence of both distance and sparingly water-soluble contact pheromones in *Lysmata wurdemanni* and *L. boggessi* [22], [23] to examine the chemical identity of the contact pheromone. The reproductive system of *Lysmata* species is rare among decapod crustaceans [24]: a shrimp first matures as a male (male phase - MP), but with growth may change into a euhermaphrodite. These euhermaphrodite-phase (EP) shrimp carry both male and female function, as such they are protandric simultaneous hermaphrodites. Subsequently, these shrimps can mate as a female during post-molt and as a male during inter-molt [24]. Male-role *L. boggessi* (either MP or EP) have an active pre-copulatory behavior (Fig. 1) that indicates these shrimp use distance pheromones to track and locate receptive females [21], [25]. However, male-role shrimp use contact pheromones to recognize newly molted (EP) reproductive female shrimp [21], thus playing a key role in intra- and inter-specific mate recognition in this species of *Lysmata*. Here, we used this distinctive behaviour to test the hypothesis that contact pheromones of *Lysmata* shrimp are cuticular hydrocarbons similar to those found in insects [5].

## Results

Can cuticular hydrocarbons (CHC's) function as contact pheromones in aquatic organisms? To investigate the chemical composition of the carapace of shrimps at different stages of their development and sexual maturity, we used solvent extractions and coated polyethylene tubes that functioned as dummy females in our bioassays with these extracts. After the extract-treated tubes were placed into the test container, shrimp did not display any search behaviour and the tubes were only recognized after being touched by the antennae or antennules. After detecting the extract-treated tubes, male phase (MP) shrimp approached these again slowly, touched, and then attempted to grasp them. For tubes treated with hexane (control), most MP shrimp did not show the grasping behavior even if they accidentally swam across the tubes (Table 1). Twenty-two of the 30 MP shrimp displayed the grasping behavior to the newly molted EP shrimp extract-treated tubes. This is significantly higher than the numbers of the MP shrimp grasping the newly molted MP shrimp extract treated tubes (4 of 30, *G*_adj_ = 23.160, *P*<0.01) and the control tubes (3 of 30, *G*_adj_ = 26.514, *P*<0.001). There was no significant difference between the newly molted MP shrimp extract and the control (*G*_adj_ = 0.151, *P*>0.05) (Table 1).

**Table 1:** Number of MP shrimp (out of 30) displaying grasping and touching behavior toward tubes treated with cuticular extracts (hexane) of newly molted euhermaphrodite-phase (EP), newly molted male-phase (MP) shrimp, and components of newly molted EP extract, including (Z)-9-octadecenamide, a blend of (Z)-9-octadecenamide and squalene (blend 1), and a blend of (Z)-9-octadecenamide and methyl linoleate (blend 2).

Sample tested	Number of grasping MP shrimp(out of 30)
EP shrimp	22*
MP shrimp	4
Control (hexane)	3
(Z)-9-Octadecenamide	14*
Hexadecanamide	4
Methyl linoleate	6
Blend 1	16*
Blend 2	19*
Control 2 (ethanol-hexane)	4

*: Number of MP shrimp displaying touching and grasping in the newly molted EP, (Z)-9-octadecenamide, and blend treatments is significantly higher (*P*<0.01) than in the newly molted MP shrimp and the control).

**Figure 2:**
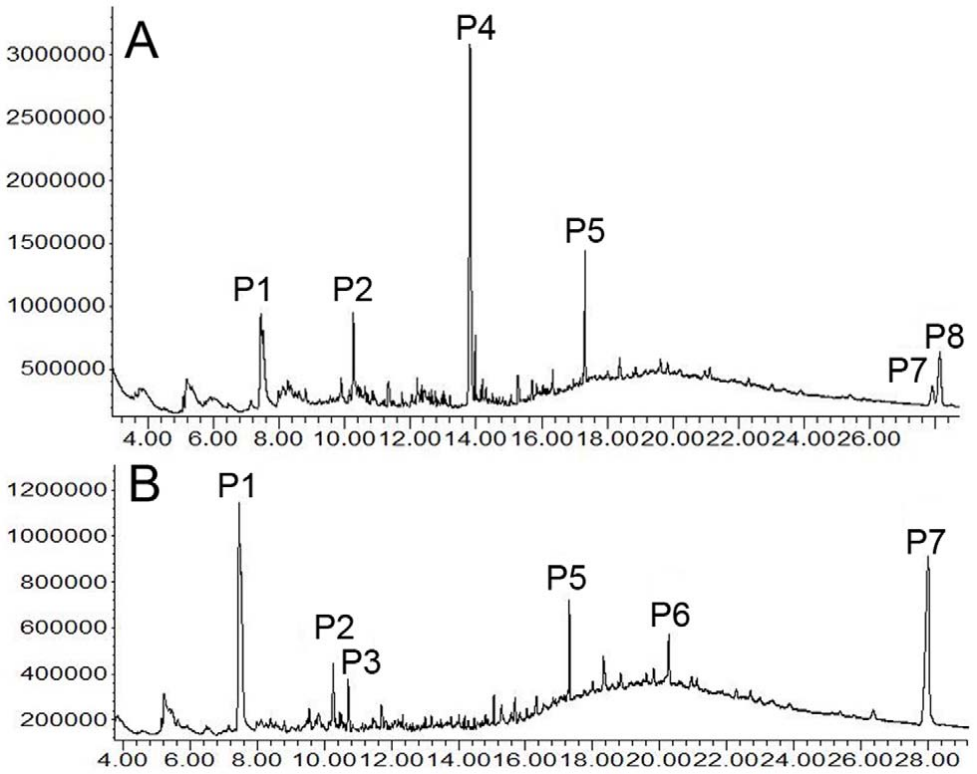
Gas chromatograms of hexane extracts of newly molted euhermaphrodite phase (EP) shrimp (top) and intermolt EP shrimp, newly molted male phase (MP) shrimp, and intermolt MP shrimp (bottom, because there are no differences between the chromatograms of the latter three samples only the newly molted MP's chromatogram is presented here). Peak 1 (pentadecyl 2-propenoate) at t = 7.44 min, peak 2 (unidentified) at t = 10.26 min, peak 3 (unidentified) at t = 10.74 min, peak 4 ((Z)-9-octadecenamide) at t = 13.81 min, peak 5 (squalene) at t = 17.32 min, peak 6 (dodecyl octadecanoate) at t = 21.30 min, peak 7 (unresolved organic acid ester) at t = 28.06 min, peak 8 (unidentified, unresolved double peak) at t = 28.16 min.

**Figure 3:**
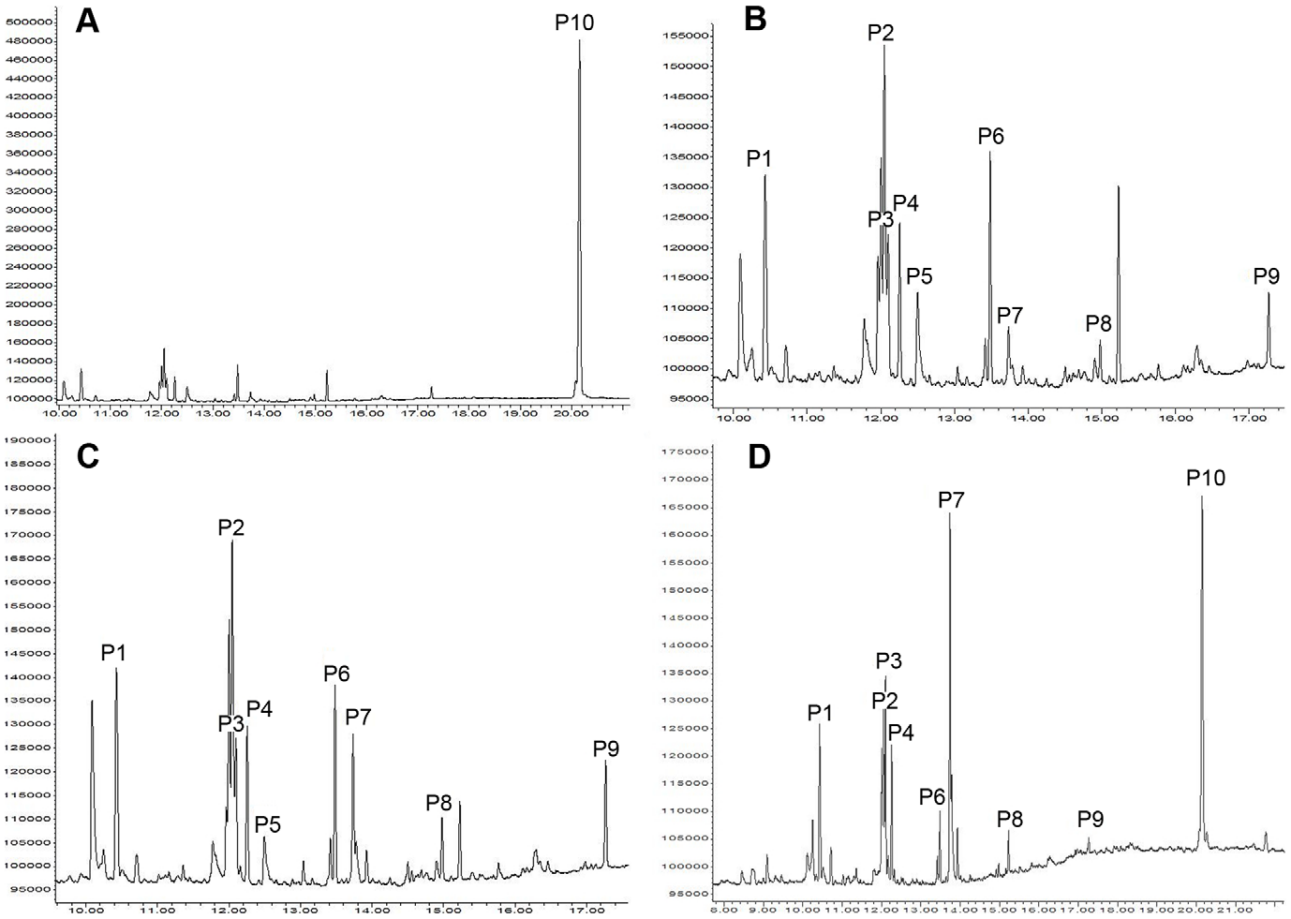
Gas chromatograms of ethanol extracts of newly molted euhermaphrodite phase (EP) shrimp and intermolt EP shrimp, newly molted male phase (MP) shrimp, and intermolt MP shrimp. A: represents the chromatograms of intermolt EP shrimp, newly molted MP shrimp and intermolt MP shrimp; B: enlargement showing detail of minor components in chromatograms of newly molted MP shrimp and intermolt MP shrimp; C: enlargement showing detail of minor components in chromatograms of intermolt EP shrimp; D: chromatograms of newly molted EP shrimp. Peak 1 (ethyl palmitate) at t = 10.43 min, peak 2 (methyl linoleate) at t = 12.05 min, peak 3 (hexadecanamide) at t = 12.10 min, peak 4 (ethyl stearate) at t = 12.25 min, peak 5 (methyl eicosapentaenoate) at t = 12.50 min, peak 6 (methyl eicopentaenoate) at t = 13.48 min, peak 7 ((Z)-9-octadecenamide) at t = 13.73 min, peak 8 (methyl or ethyl docohexanoate) at t = 14.98 min, peak 9 (squalene) at t = 17.27 min, peak 10 (unidentified) at t = 20.15 min.

We analyzed the bioactive cuticular extracts of *L. boggessi* using gas chromatography (GC) and gas chromatography-mass spectrometry (GC-MS). As Figure 2 shows, hexane-extracts of newly molted euhermaphrodite-phase shrimp (NMEP) were different from those of intermolt EP shrimps, intermolt MP shrimp and newly molted MP shrimp. There were no differences among the latter three. EP and MP shrimp shared some components, but differed in others (Table 2, Fig. 2). They have six major components in the cuticular hexane-extracts; of these, three were identified as lipophilic esters (Table 2). In cuticular ethanol-extracts, ten major components were identified as lipophilic esters (Fig. 3, Table 2). There were no qualitative differences in the chemical compositions of the ethanol-extracts of newly molted EP and MP shrimp, nor to intermolt EP shrimp. However hexadecanamide (Peak 3), (Z)-9-octadecenamide (Peak 7) and methyl linoleate (Peak 2) ratios differed between the newly moulted EP and MP shrimps. We therefore considered these three lipophilic CHC compounds as characteristic of newly moulted shrimps (EP and MP), and that their EP and MP phase specific ratios form a typical bouquet that could potentially enable shrimp to detect Euhermaphrodite-Phase (EP) animals that are ready to mate.

**Table 2:** Composition of hexane and ethanol extracts from newly molted male-phase shrimp (MP) and newly molted euhermaphrodite (EP) of *Lysmata boggessi*.

Hexane extracted cuticular components				Ethanol extracted cuticular components			
Peak	MP	EP	Compound	Peak	MP	EP	Compound
P1	+	+	Pentadecyl-2-propenoate	P1	+	+	Ethyl palmitate
P2	+	+	Unidentified	P2	+	+	Methyl linoleate
P3	+	−	Unidentified peak	P3	+	−	Hexadecanamide
P4	−	+	(Z)-9-Octadecenamide	P4	+	+	Ethyl stearate
P5	+	+	Squalene	P5	+	+	Methyl eicosapentaenoate
P6	+	−	Dodecyl octadecanoate	P6	+	+	Methyl eicopentaenoate
P7	+	+	Unresolved acid ester	P7	+	+	(Z)-9-Octadecenamide
P8	−	+	Unidentified peak	P8	+	+	Methyl docohexanoate
				P9	+	+	Squalene
				P10	+	−	Unidentified peak

+ = present, − = absent; peak numbers correlate with those shown in Figures 1 (hexane extract) and Figure 2 (ethanol extract).

Do marine shrimp use multi-component blends of CHC's to induce mating? When testing the individual bioactivity of both synthetic analogues of the shrimp cuticular extracts and authentic, isolated components from the extracts, only (Z)-9-octadecenamide elicited positive responses significantly different (2×2 G-tests with William's corrections) from the control (14 vs 4 of 30 MP shrimp, *G*_adj_ = 8.035, *P*<0.01). Both hexadecanamide (4 of 30 MP shrimp) and methyl linoleate (6 of 30 MP shrimp) were not significantly different from the control (*G*_adj_ = 0.000, *P*>0.05 and *G*_adj_ = 0.459, *P*>0.05, respectively, Table 1). Since none of these compounds individually elicited a response as potent as the complete newly molted EP shrimp extract (22 of 30, Table 1), we tested the hypothesis that a bouquet of CHC's functions as the contact cue. All blends containing (Z)-9-octadecanamide resulted in positive responses (blend with squalene, 16 of 30 MP shrimp, *G*_adj_ = 11.043, *P*<0.01; blend with hexadecanamide and methyl linoleate, 19 of 30 MP shrimp, *G*_adj_ = 16.448, *P*<0.01). Interestingly, not only were the number of shrimp responding greater, but the average time spent grasping the blend-treated tubes was also significantly longer than for the treatment with (Z)-9-octadecenamide alone, with 2.60±0.40 s for (Z)-9-octadecanamide, 2.69±0.31 s for the blend with squalene, and 6.05±1.71 s for the blend with hexadecanamide and methyl linoleate (*t*-test, *P* = 0.033, df = 31, data were log-transformed because of heterogeneity of variances).

## Discussion

Both the behavioural bioassays and our gas chromatographic analysis of extracts obtained from newly molted EP shrimp indicate that (Z)-9-octadecenamide is the major component of the CHC contact pheromone bouquet in shrimp with methyl linoleate and hexadecanamide contributing to the sex-specific bioactivity. The use of a bouquet is a common phenomenon in insect pheromone systems [4]. Although insect cuticles often contain a complex mixture of compounds, only a few may be involved in functioning as contact sex pheromones with composition or ratios varying between conspecific males and females [7], [26], [27]. Active compounds may be present in both sexes but more abundant on the cuticle of females, as shown for Asian longhorned beetles, *Anoplophora glabripennis* [28]. In these beetles, (Z)-9-heptacosene is the most abundant component within a bouquet of several other bioactive components in which a synthetic mixture still had a lower bioactivity than the authentic female extract [28].

9-Octadecenamide is a naturally occurring amide of oleic acid found in both plants and animals [29]. While our study is the first to describe (Z)-9-octadecenamide as a sex pheromone in an invertebrate, the compound has been found in anogenital gland secretions of the giant panda, *Ailuropoda melanoleuca*, where it is used to mark scent posts [30]. Both the carboxylic acid ((Z)-9-octadecenoic acid, oleic acid) as well as the saturated methyl ester (methyl (Z)-9-octadecanoate) analogues are chemical cues in a variety of insects [31]. In plants, (Z)-9-octadecenamide has been isolated from various brown algae, *Zostera marina* [32], [33], and the freshwater green alga *Rhizoclonium hieroglyphicum* [34]. In the field, *L. wurdemanni* shrimps eat such algae [35], and this makes it reasonable to suggest that (Z)-9-octadecenamide is taken up with food and then forms a component of the contact pheromone bouquet. Many pheromones are not synthesized *de novo* by animals but are either derivatives modified from precursors taken up with food or are compounds directly incorporated from food [36]. Interestingly, the two minor components of the pheromone bouquet, hexadecanamide and methyl linoleate (Table 1), are also found in algae [33].

Contact cues require limited bioavailability of odor compounds with a balance between diffusion into the environment and stability on the body surface to enable interactions with the receiver's chemoreceptors. Unlike large fatty acids, small lipophilic compounds are able to diffuse freely through water. One mechanism to increase cue stability and retention, leading to a reduction in signal production costs, is the use of matrices that function similar to slow-release gels such as the urinary proteins used in mice territorial and sexual marking [37]. The results of our study (Table 2) show that the main component of the suite of active cuticular contact pheromones of *L. boggessi*, (Z)-9-octadecenamide, is peculiar in its extractability using hexane. (Z)-9-Octadecenamide is present in the cuticles of intermolt and newly molted EP and MP shrimp when extracted with ethanol. However, hexane was effective in extracting the compound only from newly molted EP shrimp. This may be a result of either a chemical or physical difference in the body surface (i.e. chitin) that is associated with a recent molt. Molting is a dramatic rearrangement of the exoskeleton's biochemical surface properties and its physical properties. This change in the characteristics of the cuticle surface may allow (Z)-9-octadecenamide to be extracted with ethanol but not hexane. At this point in time, we can only speculate that a matrix alteration or a co-secretion (or lack thereof) of an as yet unidentified compound is the more likely reason, thus drastically changing the chemical partitioning of (Z)-9-octadecenamide between the body surface of the different shrimp forms and the hexane extraction solvent. A change in the chitin porosity or density is less likely because of the continued ability of ethanol to extract the compound. Regardless of the reason, this mechanism may be the key to the ability of male-mating shrimp to distinguish between newly molted EP (reproducing as female) and other, non-sexually responsive forms. Utilizing the physical properties of contact cues in matrices as signal delivery mechanism could be widespread amongst species where reproduction is linked to an individual's molt.

In contrast to the wealth of evidence on the role of CHC's as contact pheromones in insects, to our knowledge, the *L. boggessi* pheromone bouquet is the first direct chemical evidence of CHC's as contact pheromones in decapod crustaceans. Apart from the proposed glycoprotein sex pheromones in harpacticoid copepods, *Tigriopus japonicus* [18], only the partially surface-bound protein complex cues that control the gregarious larval settlement in barnacles, *Balanus amphitrite*, have been characterized successfully [28]. Our data support the theory that in aqueous systems, sparingly–soluble, nonpolar compounds function as contact cues as proposed originally for rotifers [38]. This further implies that the role of lipophilic cuticular hydrocarbons (CHC's) as contact cues has not exclusively evolved in insects. Contact pheromones play a key role in intra- and inter-specific recognition of many marine organisms in a large variety of behavioural contexts where direct contact of two individuals is required. This also includes symbiosis and commensalism relationships or larval settlement cues. As highlighted recently by Hay [4], future identifications of contact pheromones of other species are required to develop an understanding of contact pheromone evolution and their roles in the marine ecosystem; based on our data, it is feasible to suggest that CHC's could also play a significant role as signalling compounds in aquatic environments.

## Materials and Methods

### Ethics statement

All animal work using shrimps presented here has been conducted according to relevant national and international guidelines to ensure ethical appropriateness.

*Lysmata boggessi* shrimp were collected from Hernando Beach, Florida, U.S.A. The male phase (MP) and euhermaphrodite-phase (EP) shrimp (2.6–4.4 cm) were housed in 20-L tanks with flow-through seawater (35‰; 26–28°C), photoperiod of 14 h Day/10 h Night, and fed frozen adult *Artemia* once daily.

### Extraction of Cuticular Hydrocarbons

Based on our hypothesis that contact pheromones are non-polar hydrocarbons, HPLC grade hexane (Sigma Chemical Company) was chosen as the extraction solvent as has been previously described for insects [5], [6]. Cuticular compounds from the abdominal region of the exoskeletons of both newly molted and intermolt MP (Male-Phase) and EP (Euhermaphrodite-Phase) shrimp were extracted through immersion in 1.5 mL hexane for 30 seconds. Ten newly molted male phase (MP) or euhermaphrodite-phase (EP) shrimp, and 20–30 intermolt MP or intermolt EP shrimp were used for contact cue extractions. This was designed to provide metabolomic-type reference samples where, in the chemical analysis, we could examine differences in the chemical fingerprints (as chromatographic peaks) of these shrimps in order to earmark potentially bioactive compounds for further testing in bioassays. The pooled extracts (based on molt stage and developmental phase) were stored in 20-mL glass vials with polyethylene caps at −20°C. Prior to gas chromatographic analysis, the solvent in the samples was removed by rotary evaporation at 30°C, and the residue was re-dissolved with 1 mL hexane. The same extraction procedure was also carried out using HPLC grade ethanol (Sigma Chemical Company) as the extraction solvent.

Analytical results from the hexane extract indicated that (Z)-9-octadecenamide is a major component of the contact pheromones of *L. boggessi* (see Results). However, synthetic solid (Z)-9-octadecenamide is only sparingly soluble in hexane; as such, the (Z)-9-octadecenamide may have not have been exhaustively extracted from the shrimps using this method. Although (Z)-9-octadecenamide in the extract of intermolt EP shrimp is absent in our extracts (see Results), we assumed that (Z)-9-octadecenamide might still exist in the cuticle of shrimps but potentially in low concentrations. To examine this hypothesis, we also extracted the potential pheromones using ethanol as a solvent in order to compare the data with those achieved using hexane for extraction. The same extraction procedures were applied as those in the hexane extraction.

### Chemical Analyses

Gas chromatographic (GC) analysis of the solvent extracts of cuticular material was accomplished with a Hewlett Packard 6890 system. Eluting compounds were detected using a flame ionization detector (FID). A 2 µL aliquot of the various extracts was injected onto a fused-silica capillary column (HP-5; 30 m×0.25 mm I.D.×0.25 µm film thickness; Agilent Technologies) in split-less mode. Helium was used as the carrier gas (1.5 mL/min). The GC oven temperature was initially held at 150°C for 2 min, increased to 300°C at 10°C/min and finally held at 300°C for 20 min. Coupled gas chromatography-mass spectrometry (GC-MS) analysis of the samples was conducted also using an HP 6890 gas chromatograph system using the same chromatographic protocol described above. The GC was coupled to a HP 5973 mass selective detector and samples ionized with 70 eV using electron impact ionization (EI). Total ion count (TIC) at full scan mode (m/z range 40–600) was used to detect eluting peaks. Preliminary compound identification was made by comparing measured spectral data with mass spectra available in the NIST-library. Synthetic analogues of candidate compounds were co-injected with hexane and ethanol cuticular extracts using both the GC as well as the GC-MS systems to verify their occurrence in the natural extracts. (Z)-9-octadecenamide (Cayman Chemical Co.), methyl linoleate, hexadecanamide, and squalene (all from Sigma Aldrich) were used for this purpose.

### Behavioral Bioassays

The design of the behavioral assay [23] was to test the bioactivity of the hexane-extractable cuticular compounds. To ensure the test animals were receptive to potential sex pheromones, only MP shrimp were used and no individual was used more than once to avoid potential habituation. All bioassays were conducted blind with the observer unaware of the nature of the extracts to be tested in order to reduce observer bias. The behavioral responses of the MP shrimps were observed using fluorescent illumination and recorded with a Sony camcorder for future reference as well as independent verification of the results by a second observer. Bioassays were conducted on single MP shrimp in 10-L containers with seawater (35‰; 26–28°C). Plastic tubes 3 mm in diameter and 2 cm in length were treated with extracts (residue was re-dissolved in 1 ml hexane) from newly molted EP shrimp, newly molted MP shrimp, or hexane (as control) by immersion and then allowed to dry for 15 seconds. One tube was randomly selected and placed in the bioassay container. Thirty replicates were carried out for each treatment and the control. A positive response was scored if the MP shrimp grasped the extract treated tube, as it does when encountering a newly molted EP shrimp [24]. The number of positive responses to extract-treated and hexane-treated (control) tubes were compared using 2×2 G-tests with William's corrections for significance analysis [39].

Synthetic analogues of candidate compounds identified through GC and GC-MS of hexane and ethanol cuticular extracts (e.g. (Z)-9-octadecenamide (Cayman Chemical Co.), methyl linoleate, hexadecanamide, and squalene (all from Sigma Aldrich)) were also tested both individually as well as in blends using the above bioassay. The concentration of single compounds used was at 10^−4^ M, for blends the other compounds were added according to ratios of LS/MS spectra. Because of the low solubility of (Z)-9-octadecenamide in hexane (see above), 0.5 mg of the chemical was first dissolved in 0.1 mL ethanol and 1.4 mL hexane was then added. Blank samples containing the same ratio of ethanol and hexane were used as controls.
